# Analysis of the nutritional status in the Palestinian territory: a review study

**DOI:** 10.3389/fnut.2023.1206090

**Published:** 2023-07-18

**Authors:** Enas A. Assaf, Haleama Al Sabbah, Ayoub Al-Jawadleh

**Affiliations:** ^1^Faculty of Nursing, Applied Science Private University, Amman, Jordan; ^2^Department of Public Health, College of Health Sciences, Abu Dhabi University, Abu Dhabi, United Arab Emirates; ^3^World Health Organization Regional Office for the Eastern Mediterranean, Cairo, Egypt

**Keywords:** Palestine, nutritional status, obesity, exclusive breastfeeding, stunting, wasting, anemia, vitamin D deficiency

## Abstract

**Background:**

Food insecurity, occupation, and poverty contribute to the poor nutritional status of Palestine. This review study aimed to analyze the nutritional status in the Palestinian Territory by analyzing published data from 2011 to 2023.

**Method:**

Searching for relevant publications yielded 67 studies. Based on reviewing these studies, five major themes were identified: low birth weight, breastfeeding, obesity and overweight, protein-energy malnutrition, and micronutrient deficiency.

**Results:**

Based on the review of these studies, five major themes were identified, namely, low birth weight, breastfeeding, obesity and overweight, protein-energy malnutrition, and micronutrient deficiency. Based on the literature, the prevalence rate of exclusive breastfeeding was 24.4% in the Gaza Strip, compared to a national rate of 39.9% in 2020. Smoking, anemia in mothers, diet during pregnancy, and indoor pollution were associated with low birth weight. One-fifth of the boys and girls were stunted by 2 years of age in the Gaza Strip, and girls were more stunted than boys. The prevalence rates of underweight, overweight, and obesity among school children in the West Bank were 7.3%, 14.5%, and 15.7%, respectively. Age, gender, and living area were significant predictors of being overweight among school children. The prevalence rates of overweight and obesity among adults in Palestine were 57.8% and 26.8%, respectively. Obesity is associated with a family history, chronic diseases, and low physical activity among adults. Exclusive breastfeeding was below the WHO recommendations, while significant rates of obesity and overweight were found among children and adults. Iron-deficiency anemia (IDA) among pregnant women and children remains a challenging public health issue, while other micronutrient deficiencies are high among children.

**Conclusion:**

This review emphasizes the need for multi-sectoral interventions to address malnutrition and nutritional shifts. It identifies gaps and addresses nutrition-related issues in the Palestinian Territory, which can serve as a basis for guiding United Nations agencies and governments in formulating evidence-based policies and strategies for prioritizing nutritional interventions to meet sustainable development goals.

## 1. Introduction

Palestine (Palestinian territories) is considered one of the Eastern Mediterranean regions (EMR) that suffer from the burden of malnutrition, especially among children, involving deficiencies in micronutrients coupled with elevated rates of non-communicable diseases (NCDs), overweight, and obesity (1–3). Over the past few decades, it has been determined that unhealthy eating habits are the chief risk factor for the global burden of NCDs (3). Based on the Global Burden of Disease (2017 report), high sodium intake and insufficient dietary fibers were the two primary components responsible for 6 million deaths worldwide (4), along with an increase in the prevalence of obesity by 5.9% (5). Lifestyle change is one of the leading reasons for this increase in NCDs over the past several decades (6), leading to nutritional changes such as shifting to high-energy, saturated fat-rich, and sugar-dense meals, and decreasing consumption of complex carbohydrates and fibers (7, 8).

Malnutrition in children, particularly in the early stages of life, is considered high risk for impaired cognitive and physical growth and can increase susceptibility to infectious diseases (9–12). On the other hand, obesity among children has adverse health effects, including metabolic complications and psychological and physiological effects, in addition to long-term complications that may include premature death, NCD later in life, and disability (9, 12–14). Micronutrient deficiency, especially iron deficiency, anemia, vitamin A deficiency, and iodine deficiency, might be considered a silent emergency in many developing and low-income countries, particularly for children, which puts two billion people at risk of experiencing anemia, night blindness, and various other NCDs (15).

Palestine (the West Bank and the Gaza Strip) faces the challenges of military occupation, sieges and curfews, parental unemployment, limited food availability, poverty, and food insecurity, which have all contributed to the deterioration of the nutritional status of the Palestinian population, especially among the more vulnerable groups such as children and women (16). Combating nutrition shifts and malnutrition requires multi-sectoral and multifactorial strategies and interventions (17), particularly in Palestine. These interventions need a comprehensive review of the current nutritional status in Palestine, which would be essential in informing evidence-based prioritization of interventions, the development of national nutritional policies, and monitoring purposes. Therefore, the main objective of this review is to provide a comprehensive overview of the nutritional status in Palestine by reviewing the existing literature and research on specific nutrition indicators, including low birth weight (LBW), malnutrition, stunting, and underweight among children under 5 years of age, as well as breastfeeding practices, overweight and obesity among both children and adults and related behaviors. Additionally, the review focuses on micronutrient deficiencies, including iron deficiency anemia trends among women of reproductive age and children, as well as deficiencies in vitamin A, vitamin D, and iodine-based on the available data and studies. This study's recommendation can aid in the development of policies and strategies that can serve as a guide and tool for donor organizations and government entities to prioritize interventions and formulate nutritional strategic plans. Moreover, this study will shed light on critical data gaps and emphasize the need for political support and interest from United Nations agencies. Furthermore, it aims to address the recommendations of the International Conference on Nutrition (ICN)-2, tackle NCDs, work toward global targets for nutrition, and contribute to the achievement of Sustainable Development Goals.

## 2. Materials and methods

### 2.1. Palestinian background information

The estimated population of Palestinian territory (West Bank and Gaza Strip) is 5.35 million (3.18 million in the West Bank, including east Jerusalem, and 2.17 million in the Gaza Strip) (18), and 44% of the population are children (19). Palestine is a densely populated country, with 781 inh/km^2^ (the Gaza Strip is 5,138 inh/km^2^, and the West Bank is 500 inh/km^2^) (20). The estimated life expectancy in 2020 was 74.1 years (West Bank: 74.4 years, Gaza Strip: 73.7 years) (women: 75.3; years, men: 73.3 years) (21). The mortality rate for those under 5 years was 14 per 1,000 (boys: 16 per 1,000, girls: 12 per 1,000) (22). In 2020, NCDs accounted for more than two-thirds of Palestinian deaths (23). Concerning food insecurity, the report from 2020 indicated that 40% of households in the West Bank and 60% in the Gaza Strip are moderately to severely food insecure (24). Moreover, the poverty rate in the Gaza Strip was 53%, compared to 13.9% in the West Bank and 29.2% at the national level (25).

### 2.2. Study review methodology

To analyze the nutritional status in the Palestinian Territory, a search was carried out between November 2022 and March 2023 to identify relevant studies published in the English language, utilizing various scientific databases, such as PubMed, Science Direct, Scopus, Google Scholar, the ResearchGate website, the Palestinian Ministry of Health website, the Global School-Based Student Health Survey (26), the Palestinian Central Bureau of Statistics (PCBS), and the World Health Organization Seventy-Fifth World Health Assembly A75/26 Provisional Agenda Item 20 (27). Additionally, data from WHO databases, such as the WHO/UNICEF Joint Child Malnutrition Estimate 2021 (28), the WHO/Nutrition Country Profile (29), and the STEP-wise Approach to NCD Risk Factor Surveillance (STEPS) 2021, were also accessed and reviewed (30).

This review presents the prevalence and trends of various nutritional indicators, focusing on the periods when national data are available over the years. Moreover, specific national indicators were evaluated based on the WHO target goals for 2025 (31). The search terms used in combination included; “malnutrition” OR “obesity” OR “stunting” OR “under-nutrition” OR “micronutrient deficiency” OR “nutrition status” OR “diet-related risk factors” OR “national nutrition strategy” OR “nutrition government policy” OR “nutrition health policy” AND “Palestinian Territory” Or “West Bank” OR “Gaza Strip” OR “State of Palestine.” The search was limited to articles published from January 2011 to January 2023. Selected sources included journals, books, master's and PhD theses, book chapters, and government data sets. Magazine and newspaper articles were not used as sources for this analysis. A total of 67 studies were extracted using the above filters and keywords.

**Figure 1:**
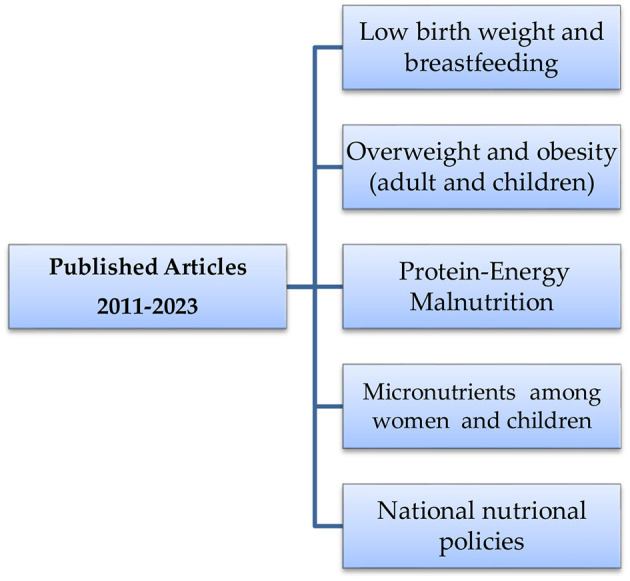
Literature review's main themes.

After reviewing all relevant literature, the following five main themes were created according to the co-author's recommendations (Figure 1):

1. Low birth weight, exclusive breastfeeding, and complementary feeding.
2. Obesity and overweight by age groups.
3. Protein and energy malnutrition (stunting, wasting, and underweight).
4. Micronutrient deficiencies.
5. National nutritional policies in Palestine.

## 3. Results

### 3.1. Low birth weight, exclusive breastfeeding, and complementary feeding

#### 3.1.1. Low birth weight

**Figure 2:**
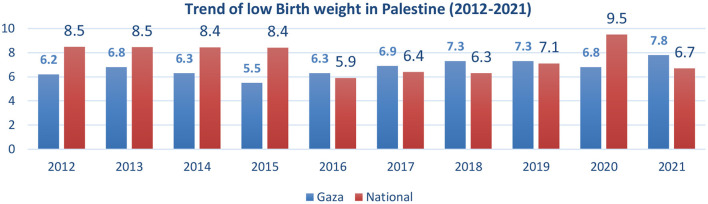
Trends of low birth weight (Infant Weight <2,500 gm) in the Gaza strip and the national Palestinian data between 2012 and 2021. Sources: UNICEF/WHO: data.unicif.org, who.int/nutgrowthdb/LBW estimates, State of Palestine, Ministry of Health yearly report of 2016, 2017, 2018, 2019, 2020, and 2021. Health Information Center, Palestinian Ministry of Health/Gaza Strip Indicators Report 2016–2020, Health Indicators for Gaza Strip 2020–2021 (41–51).

Overall, nine studies were conducted (five were conducted in the Gaza Strip, two in the West Bank, and two were national studies) (32–40). The prevalence of low birth weight, as reported by the Palestinian Central Bureau of Statistics, was 10.7 nationally (11.8 West Bank, 9.1 Gaza Strip) between 2019 and 2020 (41). A study indicated that exposure to war and occupation in Gaza is associated with an increased prevalence of LBW (40). Another factor is exposure to indoor pollution from tobacco smoke and wood fuel smoke (33). The prevalence rate of LBW reported by Al Natour and her colleagues was 15.1% in a northern city of the West Bank (32), while smoking, anemia in mothers, parity, and diet during pregnancy were found to be associated with LBW. Diet was also discussed in another study in Gaza, as specific diets for pregnant women (an Asian-like pattern) that consist mainly of vegetables, beans, and a less fatty diet were more protective against LBW (34). The trend of LBW shows a decline in the percentage between 2012 and 2021 from 8.5% to 6.7% (Figure 2).

#### 3.1.2. Exclusive breastfeeding

**Figure 3:**
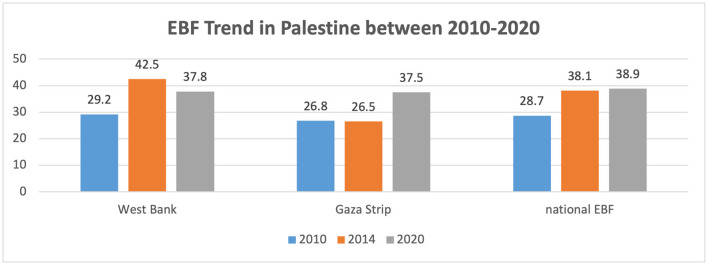
Trends of exclusive breastfeeding in Palestine between 2010 and 2020. Source: UNICEF, UNICEF Data: Monitoring the Situation of Children and Women (52) note: not all regions were included in the West Bank or the Gaza Strip based on the available data.

Nine studies discussed breastfeeding in Palestine: four in the Gaza Strip, five in the West Bank, and one national (53–61). The reported prevalence rate of exclusive breastfeeding in the Gaza Strip was 24.4% (56), whereas the reported national EBF (52) in 2020 was 39.9%. However, data from the Palestinian Central Peru of Statistics (PCPS) for the year 2019–2020 indicate that the total EBF was 43.3% (41.9% West Bank, 44.8% Gaza Strip) (62). However, it was addressed in the studies conducted in the West Bank and Jerusalem that employed mothers were less likely to practice exclusive breastfeeding than unemployed mothers. The perception that breast milk is insufficient to meet the infant's needs, along with factors such as the mother's age and the number of children, serve as determinants for exclusive breastfeeding (53, 54, 60). Figure 3 shows the trend of the national EBF (0–5 months) from 2010 to 2020, demonstrating that the EBF increased from 28.7 to 38.9.

#### 3.1.3. Complementary feeding

Only two studies were found related to complementary feeding without specifying the type and frequency of complementary feeding; one study in Gaza found that more than half (55%) of women imitated commentary feeding between 4 and 5 months (63). Another study in Gaza investigating weaning practices among infants younger than 2 years found that the majority of women initiate complementary feeding at < 6 months (64). One report from UNICEF regarding the nutritional status of children reported that among Palestinian children, only 42 % receive a minimum diversity diet (this includes receiving food from at least four of the seven nutritional groups: (1) legumes and nuts; (2) grains or roots; (3) milk products; (4) Flesh food like meat, liver, and fish; (5) eggs; (6) vegetables rich in vitamin A; and (7) other vegetables and fruits.

### 3.2. Protein energy malnutrition (stunting, wasting, and underweight) among children under 5 years

No studies were conducted in the West Bank; however, seven studies were conducted in the Gaza Strip (16, 65–70). Tsigga and Grammatikopoulou (70) found in their review study that the trend of the prevalence of underweight and wasted children in the Gaza Strip slightly declined after 2004. Al Balbesi and his colleagues (65) found that stunting was observed in one-fifth of boys and girls by 2 years of age, and girls were more stunted than boys. According to a study by El-kishawi et al., short maternal stature and parental consanguinity were factors associated with stunting in the Gaza Strip (67). Other risk factors found by Al-Najar et al. (69) were poor awareness of healthy diets, poverty, poor socioeconomic situations, urbanization, and lifestyle among communities. Moreover, the political situation and the blockade in the Gaza Strip are associated with all the previously mentioned risk factors.

**Figure 4:**
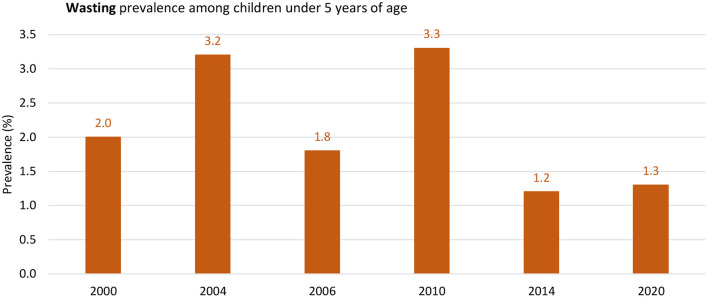
The trend of Wasting among Children Under 5 years old in Palestine between the year 2000 to 2020. Source: WHO Global Health Observatory, The UNICEF/WHO/WB joint child malnutrition estimates for stunting and overweight (71).

**Figure 5:**
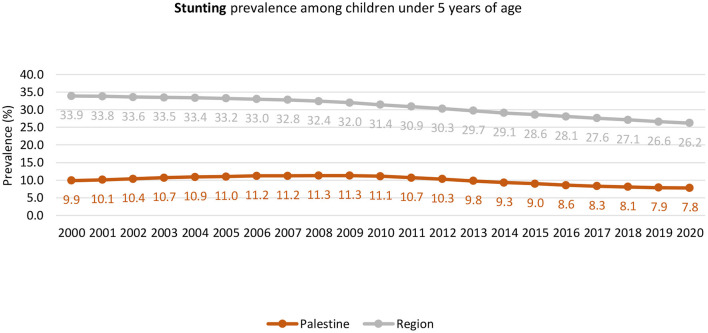
Trend of stunting among children under 5 years old in Palestine between the years 2000. Source: WHO Global Health Observatory, The UNICEF/WHO/WB joint child malnutrition estimates for stunting and overweight (69).

The household study found an interesting result related to food security and nutrition knowledge and attitudes, as more than half of food-insecure households have inadequate nutrition-related knowledge and negative nutrition-related attitudes (77.6%), and close to all of the studied sample (95.2%) did not achieve a minimum dietary diversity score (68). The reported prevalence of growth indicators at 12 months of age at primary health care centers in 2021, based on the annual health report from the Ministry of Health, was as follows: 0.4% stunting, 0.3% underweight, and 0.2% wasting (50). The WHO presented trends in waste prevalence for children under 5 years old in Palestine from the year 2000 to 2020 to show that it decreased from 2 to 1.3% (Figure 4). However, the trend shows a slight decrease in stunting prevalence from 2000 to 2020, which is less than the global prevalence (Figure 5).

**Figure 6:**
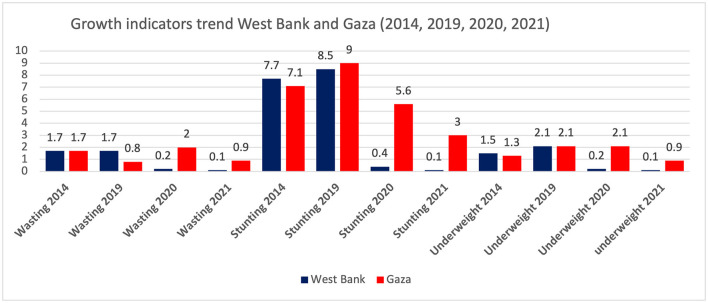
The trend of wasting, stunting, and underweight among children under 5 in the West Bank and Gaza strip in years 2014, 2019, 2020, and 2021. Sources: PCPS, Palestinian Multiple Indicator Cluster Survey 2019-2020, Survey Findings Report, Palestinian Multiple Indicator Cluster Survey 2015, Survey Findings Report, Ministry of Health Yearly Report 2021, Ministry of Health/Gaza Strip, Health Indictors, 2021 (41, 49, 50, 72, 73).

In relation to the prevalence of underweight in Palestine, Figure 6 shows the trend of underweight in the West Bank and Gaza Strip between 2014 and 2021, where it shows a decline in both areas (West Bank: 1.5–0.1, Gaza Strip: 1.3–0.9). One study by El Kishawi and her colleagues discussed the dual form of malnutrition in three areas in the Gaza Strip, which was conducted as a household study measuring the Body Mass Index (BMI) for mothers of underweighted classified children to determine that the dual form of malnutrition was 15.7% in the Gaza Strip. However, low monthly income, low level of father education, low level of maternal nutrition knowledge, and birth order were all found to be risk factors (74).

### 3.3. Obesity and overweight by age groups

#### 3.3.1. Overweight and obesity and underweight among children

There were 13 studies found in both the West Bank and the Gaza Strip (75–87). In the West Bank, one study was conducted in the northern region among school children. The study revealed that the prevalence of overweight and obesity among children was 14.5% and 15.7%, respectively. The main predictors for being overweight were age, gender, and living area (75). Another study conducted by Ghrayeb et al. (76) in the southern area of the West Bank among school children found that the prevalence of overweight was 18.6% and obesity was 9.2%. Interestingly, a study conducted in the West Bank in 20 marginalized schools found that the overall prevalence of underweight, overweight/obese students was 6% and 34%, respectively. However, the prevalence of overweight and obesity differed between 6th-grade and 9th-grade students. In 6th grade, the prevalence was 43% for boys and 24% for girls, whereas, for 9th-grade students, it was 20% for boys and 42% for girls. Factors that were found to be significant contributors to this difference included 9^th−^grade students consuming fewer milk products, engaging in less physical activity, consuming more sugar in their diet, and consuming more carbonated soft and energy drinks (78, 79). A study conducted by Massad and his colleagues (80) in 22 UNRWA schools in the West Bank found that the prevalence of overweight was 12% and obesity was 6%. Factors associated with being underweight were male sex, unemployed mothers, and households not having enough food for at least the last 2 days, whereas older age, long time spent watching TV, and low physical activity were found to be associated with being overweight. A study in the West Bank found that those who were not exposed to any form of violence and had good health literacy were less likely to be obese (83).

Concerning underweight studies among children more than 5 years old, only two studies were found; the first one conducted in Nablus city (73), the Northern region of the West Bank, found that the prevalence rate was 7.3%, and the second one was in Jerusalem among school-age children, as it found that 4.8% were underweight and/or anemic (23.3%) (84).

**Figure 7:**
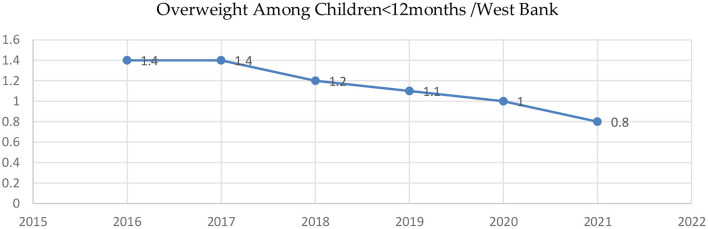
Trends of overweight among Children <12 months in the West Bank, Palestine. Sources: State of Palestine, Ministry of Health MOH, health annual reports (2016-2022) (45–50).

Studies that correlate obesity among children in Palestine with hypertensive disorders, diabetes mellitus, leptin, and lipid profiles have found a strong relationship between being overweight and obese (77, 82, 86). In one household study involving children under 5 years, the prevalence of overweight/obesity in both the West Bank and the Gaza Strip was 8.8% (7.3% overweight and 1.5% obese) and 1.4% underweight. The prevalence was higher in the West Bank than in the Gaza Strip among the wealthiest households and boys (81). However, the prevalence of overweight reported by the Ministry of Health (MOH) in the West Bank among children under 12 months old in 2021 was 0.8% (50). The trends based on the MOH annual health report show a decrease from 1.4 to 0.8% in 2021 (Figure 7).

**Figure 8:**
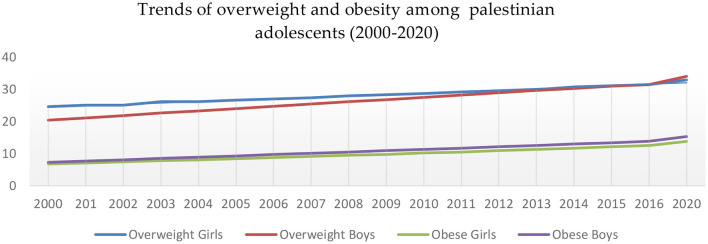
Trends of Palestinian adolescents overweight and obesity between 2000 and 2020. Source: Global Nutrition Report, Country Profile of the State of Palestine (88).

The trend of adolescent overweight shows an increase from 2000 to 2020 (from 20.4% to 34% in boys and 24.6% to 32.9% in girls). In addition, obesity shows an increase in both girls and boys (from 6.8% to 13.8% and 7.3 to 15.3, respectively) based on the Country Nutrition Profile report (Figure 8) (88). One study conducted in Hebron city in the West Bank discussed the prevalence and psychosocial impact of obesity among adolescents, finding that the prevalence of obesity was 3.3% and overweight was 13.8%, and finding a high significance between obesity and low student self-satisfaction (87).

#### 3.3.2. Obesity among adults

Overall, 16 studies were found to be related to obesity among adults within the studied time frame (8 studies in the West Bank, two studies in the Gaza Strip, and five national studies) (89–104). Abdeen and his colleagues found that the prevalence of overweight in the West Bank was 35.5% among women and 40.3% among men, while obesity was 31.5% in women and 17.5% in men (89). Two studies involving mothers in the Gaza Strip found that the prevalence of overweight and obesity was 64.1%, and among urban and refugee populations, it was 67.5%. In addition, there were significant associations with age, medium and high education, high household income, nutritional education, and non-working women (90, 91). One study was conducted among university students in the West Bank to find the prevalence of overweight and obesity at 25% (31.1% men, 15.6% women) and 7.2% (9.4% men, 4% women), respectively. Moreover, it was associated with a family history of obesity and low physical activity. The study also found that 27.1% of the participants were pre-hypertensive (93). Another study among female university students in the West Bank found the prevalence of overweight and obesity at 12.4% and 1.7%, respectively (102). The available national data based on the STEPS Survey conducted in Palestine between 2010 and 2011 showed that the prevalence of overweight among adults was 57.8%, while obesity was 26.8% (105).

Studies have found that obesity among adults in Palestine and hypertensive disorders, diabetes mellitus, and cholesterol levels were highly correlated (92–96, 98, 103). In one study, waterpipe smoking was also strongly associated with increased BMI (99). The impact of COVID-19 was also studied in terms of decreasing physical activity, increasing dietary intake, and smoking, which is significantly associated with increasing BMI among Palestinians (97, 100, 104). One study discussing the effect of obesity during pregnancy and its consequences found that among pregnant women with class III obesity, 5% suffered from hypertensive disorders, and 13.9% delivered large babies (101). The prevalence of obesity during pregnancy was reported by the Ministry of Health/Gaza Strip health indicators in 2020 and 2021 as 18.9% and 25%, respectively (43, 44).

### 3.4. Micronutrient deficiency

#### 3.4.1. Iron deficiency anemia

##### 3.4.1.1. Iron deficiency Anemia among women

According to a study conducted in the Gaza Strip, the prevalence of anemia was 20.7% in the first trimester and 42.8% in the second and third trimesters. Serum ferritin levels were 23.6 in the first trimester and 38.6 in the second and third trimesters (106). A study conducted in Hebron, West Bank, found that the prevalence of IDA was 25.7% and about half of them (52%) had depleted iron stores and experienced serious pregnancy consequences for those women, including low birth weight and the frequency of preterm labor (107).

**Figure 9:**
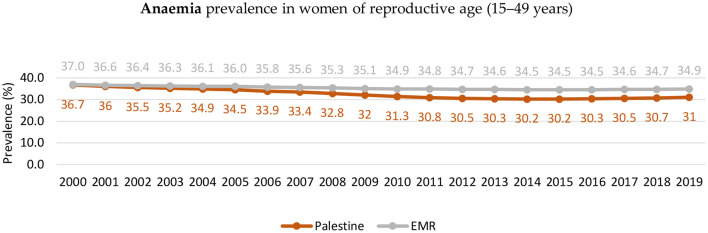
Trends in anemia among women in reproductive age in palestine compared to the global prevalence (2000–2019). Source: WHO Global Health Observatory (109).

**Figure 10:**
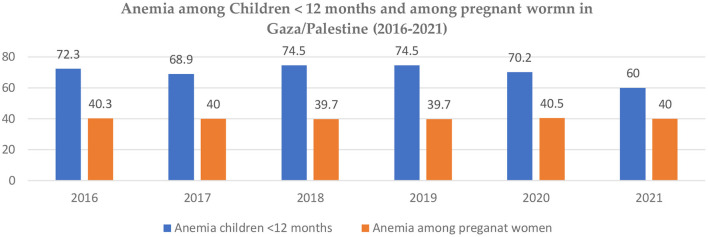
The prevalence of anemia among children less than 12 months and anemia among pregnant women in the Gaza Strip (2016–2021). Source: Ministry of Health/Gaza Strip, National Health Indicators (2016–2021) (44, 73).

In the Gaza Strip, the prevalence of anemia among secondary female students older than 15 years was 33.5%. The main risk factors for anemia were skipping breakfast, eating 1–2 meals daily, the father's job status, the average monthly expenditure, a sedentary lifestyle, and the duration of menstruation (more than 7 days). Anemia was also strongly correlated with poor academic performance (108). The reported anemia among women of reproductive age decreased from 36.7 in 2000 to 31 in 2019 (based on the WHO Global Health Observatory, Figure 9) (109). Figure 10 shows the prevalence of anemia among pregnant women in the Gaza Strip between 2016 and 2021 (based on the Ministry of Health's national health indicators 2016–2021) (43, 73), which looks relatively stable but high.

##### 3.4.1.2. Iron deficiency anemia among children

A study in the Gaza Strip conducted to assess the level of anemia among preschool children found that the prevalence was 59.7% (46.5% mild and 13.5% moderate). Factors found to be significant were the area of living, boys being more susceptible, poor households, and being underweight (110). A kindergarten study in Gaza found a prevalence of 33.9% iron deficiency anemia, and the main associated factors were the area of living and a low level of parental education (111). Jalambo and his colleagues [111] found the prevalence of anemia, stunting, and parasite infection in Gaza among 5–6-year-old children at 40.7%, 9.1%, and 17.1%, respectively. A study in the Gaza Strip among adolescent female students found that the prevalence of anemia was 35.8%, iron deficiency was 40.3%, and skipping breakfast, the amount of junk food intake, low consumption of fruits and vegetables, and mothers' education were all found to be associated factors (112). A KAP study among adolescents in Gaza found that 81.3% were not aware of the consequences of IDA among pregnant women, 89% were not aware of iron-rich foods, 74.8% were not aware of foods that reduce iron absorption, 81.7% usually consumed tea or coffee, and more than half consumed them daily. In addition, two-thirds were unsure or did not consider IDA a significant condition (113).

Qasrawi et al. (114) found in their study in the West Bank that both boys' and girls' school achievements were highly associated with the adequacy of fruits and vegetables and a low intake of soft drinks and energy drinks. Figure 10 shows the prevalence of anemia among children younger than 12 months between 2016 and 2021, which indicates a decrease from 72.3% to 60% based on the Ministry of Health's Gaza Strip health indicators (44, 73).

#### 3.4.2. Other micronutrients

The 2013 Palestinian Micronutrient Survey reported the prevalence of the following deficiencies among pregnant women in the first trimester in the West Bank and Gaza Strip: 21.3% and 23.6% for iron, 49.6% and 67.9% for zinc, 8.8% and 11.4% for vitamin A, 66.7% and 78.6% for vitamin D, 19.1% and 27.9% for vitamin B12, and 13.2% and 17.5% for vitamin E, respectively, and those rates show higher for the second and third trimesters (115).

One study was conducted in the West Bank by UNRWA and governmental schools to assess the level of micronutrients post-national interventions at grades six and nine for female and male students and found that the prevalence of micronutrients was low: iron; 9.6%, MCV; 18.8%, folate; 2.4%, zinc; 31.3%, B12; 22%, thyroxine; 8.2%, and thyroid stimulating hormone; 3.6%. The study also found that gender differences were more prevalent among girls with iron deficiency anemia, area of living, and type of school (UNRWA schools had a higher prevalence compared to governmental schools) (116).

Horino et al.(106), in their study on micronutrient deficiencies in the Gaza Strip among pregnant women, reported the following findings:

- Zinc deficiency was observed in 67.9% of pregnant women during the first trimester, while it increased to 84.7% during the second and third trimesters.
- Folate deficiency was found in 2% of pregnant women during the first trimester, which rose to 10% during the second and third trimesters.
- Vitamin A deficiency was identified in 11.4% of pregnant women during the first trimester and increased to 18.6% during the second and third trimesters.
- Vitamin B12 deficiency was observed in 27.9% of pregnant women during the first trimester, and it rose to 51% during the second and third trimesters.
- Vitamin D deficiency was prevalent in 78.6% of pregnant women during the first trimester, while it decreased to 69% during the second and third trimesters.

Another study on micronutrient deficiencies among lactating women found that 2.7% in the West Bank and 19.7% in the Gaza Strip had iron deficiency anemia, 88.8% in the West Bank and 92.7% in the Gaza Strip had Zinc deficiency, 36% in the West Bank and 24% in the Gaza Strip had vitamin D deficiency, and 33.1% in the West Bank and 92.7% in the Gaza Strip had vitamin A deficiency. A significant association was found between those who consume chocolate and the type of community (117).

A study regarding nutrient intake and adequacy among preschool children in the Gaza Strip found that 75% consume less than the recommended dietary allowance. The highest level of deficiencies was found in energy (89.8%), followed by calcium (73.3%), iron (47.2%), carbohydrates (20%), and Zinc (17%) (118). A study assessing the risk factors for vitamin A and D in the West Bank and Gaza found that the overall national prevalence was 73.1% and 60.7%, respectively. The main risk factors highlighted were that children in Gaza had a higher prevalence of 1.34 and 1.96 times than in the West Bank, older children were more susceptible, and female children were more susceptible (119). Concerning iodine deficiency, a study conducted in Jenin, West Bank, among pregnant and lactating women and newborns tested the levels of iodine in breastmilk, pregnant women's urine, and infant blood samples, to find out that the levels were below the WHO epidemiologic criteria and optimal level of iodine, considering that newborn iodine levels will be affected by the mother's breast milk. This was explained in the study by the low intake of iodine (120). However, iodine intake is considered sufficient based on the WHO observatory and trends in iodine levels in the Eastern Mediterranean Region (121).

### 3.5. Palestinian national nutrition policies

Policies and strategic plans in Palestine have been reviewed and summarized in Table 1.

**Table 1:** List of national policies and strategic plans available in Palestine.

**Policy/program/strategic plan**	**Year of implementation**	**Status**
Policy on salt iodization	2005	Active
Wheat flour fortification	2006	Active
Child growth monitoring	2010	Active
Code of marketing of breast milk substitutes	2012	Active
Strategy or plan of action on infant and young child feeding	2017–2022	Achieved
Development of a national nutrition strategy or action plan	2017–2022	Done
Plan of action for obesity prevention	2017	Active
Policy to reduce salt/sodium consumption	2019–2020	Active
Policy to limit trans-fatty acids intake	2021	Active

Source: Policies in Palestine: In Global Database on the Implementation of Nutrition Action (122).

## 4. Discussion

### 4.1. Low birth weight and exclusive breastfeeding

#### 4.1.1. Low birth weight

Our review showed a decrease in the trend of low birth weight in Palestine, which had decreased between 2012 and 2021 (8.5%−6.7%). However, in the Gaza Strip, the percentage is still relatively high (9.7%). While the study in the West Bank has a different prevalence (13.7%), this rate was below the global level (14.6%) (123), not far from Western Asia (10.9%) (124) but not less than that in the UAE (11.8%) (125), and Jordan (16.7%) (126). Factors discussed in the studies were smoking, anemia, a high number of parities, and diet during pregnancy. This was consistent with other studies in Nepal, Ethiopia, and Brazil, with comorbidity and low iron intake during pregnancy (127–129). Another study in the UAE by Taha and her colleagues found other factors included cesarean section delivery, preterm birth, and first-child orders (130).

#### 4.1.2. Exclusive breastfeeding

Several studies discussed the prevalence rate of exclusive breastfeeding in both the West Bank and the Gaza Strip. The prevalence in overall Palestine (43.3%) was below the WHO target prevalence and other regional countries, such as the reported prevalence in the UAE (59.7%) (131) but higher than those in Jordan (25.4%) (126) and Lebanon (27%) (132). Factors associated with low exclusive breastfeeding found in both the West Bank and the Gaza Strip include the perception that breast milk is not sufficient to meet an infant's needs, a younger maternal age, and the number of children. These factors were mainly addressed in studies conducted in the West Bank and Jerusalem, particularly among employed mothers. These findings are consistent with global trends and with observations from various other studies (133–135). Previous studies have shown that early nutrition and breastfeeding might play a significant role in maintaining immunity, preventing non-communicable diseases, and promoting cognitive and physical growth (136, 137). However, several barriers discussed in global studies that minimized breastfeeding rates and facilitated mixed feeding were the lack of designated breastfeeding facilities in working places, shopping malls, and airport communities, as well as the perceived community attitudes toward breastfeeding and formula feeding that were enhanced by milk companies and the market (137–140).

### 4.2. Protein energy malnutrition (stunting, wasting, and underweight) among children under 5 years

This research area was studied extensively in the Gaza Strip but not in the West Bank; it could be related to the fact that many NGOs and humanitarian organizations, together with UNRWA, are working in the Gaza Strip more to assess the nutritional status under the blockade of the political changes. Children's nutritional status and rights are crucial since they could be affected. Therefore, monitoring the malnutrition status in the Gaza Strip was more than in the West Bank. Based on the national data, wasting, stunting, and being underweight have dramatically decreased since 2014. However, the review showed differences between the West Bank and the Gaza Strip. In Gaza, the percentage is still high compared to the West Bank. Wasting in Palestine was reported to be 1.3%, which was below the global (6.7%) and regional (5.1%) levels (141) and the prevalence reported in Jordan (2.4%) (126). The prevalence rate of the national data was also below the regional level (71). Factors identified in Gaza studies were parental consanguinity, short maternal stature, gender, poor awareness of healthy diets, poverty, poor socioeconomic situations, urbanization, and lifestyle, in addition to the political blockade in Gaza. Similar to what was found in African studies and Afghani refugees (14, 142), other studies found short paternal height, socioeconomic factors, and parental educational level (143).

Among the interesting studies was the study by El Kishawi and her colleagues, which found a strong relationship between dual malnutrition of the mother and her child, as the percentage of malnutrition was 15.7% in Gaza, where low-income and parental education together with birth order were found to be associated factors. Considering that poverty affects both the mother's and the child's health, this alarming situation may be a strong indicator of poverty in the Gaza Strip. Therefore, breastfeeding and other nutritional strategies must consider the parent's educational level and poverty status. Dual malnutrition was found in other studies, particularly in Africa, where the factors listed were much more similar (144).

### 4.3. Obesity and overweight

#### 4.3.1. Children obesity

There are a greater number of studies discussing childhood obesity in the West Bank compared to the Gaza Strip. These studies have revealed that the prevalence of childhood obesity varies depending on factors such as geographical area, type of school, family wealth, and gender. The prevalence of overweight and obesity in 2020 data was higher among boys than girls (34%, 32%), (15.3%, 13.8%). The overall obesity level exceeded the global prevalence of (5.7%) in 2020 (145) and in Jordan, (6.5%) (126), and was very close to the UAE as one of the developed gold countries in both overweight and obesity prevalence of 35.8% and 17.3%, respectively (125). Childhood obesity is a key predictor of future health and the development of chronic diseases.

Three studies collating the BMI among children with metabolic disorders and hypertension found interesting results, similar to the study findings by Aburawi et al. (146), where they found that children with excess fat had increased risks of developing dyslipidemia, systemic inflammation, cholestasis, endothelial dysfunction, and diabetes. Obesity among children is considered one of the most alarming public health risks and problems since it may lead to various psychological and physical complications (147). Therefore, it is crucial to focus on more studies on the Gaza Strip children's problems, owing to the fact that poor nutritional habits, particularly high-fat and high-carbohydrate diets, might lead to overweight and obesity, which can be accompanied by malnutrition from both carbohydrates and fats used previously to build a dietary weight loss regime (148). Factors that were found to be associated with childhood obesity in Palestine and were consistent with those in other studies included consuming fast food and sugar-sweetened beverages (149, 150), low physical inactivity (151), age, as more obesity was found in adolescence rather than younger age (152), and time spent on TV (153, 154), and boys were more prone to being obese in comparison to girls (152). COVID-19 lockdown was also found to affect children's eating habits, as what was found in Rome has a negative influence on children in terms of eating habits and sedentary life with increased childhood obesity (155).

#### 4.3.2. Adult obesity

The prevalence of obesity among adults in Palestine, either in the West Bank or Gaza Strip, found a high prevalence of overweight (more than half of the participants) and obesity (about one-third of the participants), which is considered a real problem that requires attention and the formulation of health policies and strategies. Factors mentioned as being associated with overweight and obesity among adults in Palestinian studies were the following: lifestyle changes such as decreased intake of fruits and vegetables, consumption of caloric beverages, snacking, a lack of physical activity, and smoking were all consistent with studies in different countries regionally and globally (150, 156–159). Obesity was more prevalent among women than men in Palestine due to cultural constraints, lifestyle, and low physical activity, which was similar to what had been found in different studies compared to men (160). Interestingly, several studies have examined the connections between obesity and hypertension, diabetes, and cardiac diseases among adults. These studies have shed light on the impact of obesity on a person's overall quality of life (161–163). One study in the West Bank examined the prevalence of obesity among pregnant women and pregnancy outcomes. Their results were highly consistent with those of other studies that found a strong relationship between induced pregnancy hypertension and delivering large babies (164, 165).

### 4.4. Micronutrients

#### 4.4.1. Iron deficiency anemia

Iron deficiency anemia (IDA) is a public health problem and was ranked number nine among the modifiable risk factors for death (166). Several studies were reviewed regarding IDA among women of reproductive age in both the West Bank and the Gaza Strip; the prevalence based on the WHO (30%) was found to be close to the regional level (34.9%) (167). However, data from the Ministry of Health/Gaza indicators was 40% higher than the regional prevalence (44). In Palestine, the prevalence of iron-deficiency anemia (IDA) among pregnant women remains a challenging public health issue, with a rate higher than that of the UAE (24.3%) (125) but lower than that of Jordan (43%) (126). One study identified IDA among non-pregnant women, with risk factors including skipping breakfast, consuming only 1–2 meals per day, father's job status, average monthly expenditure, personal monthly expenses, sedentary lifestyle, and prolonged menstruation (more than 7 days). Furthermore, the study found that anemia was highly associated with poor academic performance, a trend observed in other studies (168, 169).

Although the trend of anemia among children < 12 months in the Gaza Strip has shown a decline, it remains unacceptably high. Further attention is required to identify the underlying causes and associated factors to enable the planning of more effective actions (170). The prevalence of iron-deficiency anemia (IDA) in Palestine is considerably higher than that of Saudi Arabia (51%) (171) and the UAE (29.9%) (125). Literature has demonstrated that IDA has a detrimental effect on the physiological and psychological wellbeing of school-age children and their academic achievement. These findings are consistent with those of other studies (172, 173). Skipping breakfast, consuming excessive amounts of junk food, having a low intake of fruits and vegetables, and mothers' low levels of education are found to be associated with iron-deficiency anemia (IDA) among kindergarten and school-age children. These risk factors are similar to those in the UAE and Saudi Arabia (125, 171). Given these findings, there is a need for targeted programs that educate children and families on the importance of a healthy diet and proper nutrition to reduce the prevalence of IDA among children.

#### 4.4.2. Other micronutrients

Micronutrient deficiency is prevalent in national and local studies in the West Bank and the Gaza Strip, including zinc, vitamin D, and vitamin A, in pregnant women and children. Zinc deficiency was among the highest in both women and lactating mothers. In a population study, the percentage of pregnant women with zinc deficiency in South Asia ranges from 15 to 74% (174). A study in Ethiopia found that the associated factors were increased coffee intake, low animal-source diets, and a lack of diet diversity (175). Another study in Jordan found a significant association between zinc deficiency among pregnant women and pre-eclampsia (176). In Palestine, further studies are recommended to address more than micronutrient deficiency among children and pregnant women.

## 5. Conclusions and recommendations

In conclusion, low birth weight (LBW) remains a problem in Palestine, with rates ranging from 8.5% to 6.7% from 2012 to 2021. Exposure to war and occupation, indoor pollution, smoking, anemia in mothers, and a poor diet during pregnancy are risk factors associated with LBW. Exclusive breastfeeding rates in Palestine have improved in recent years, with national rates reaching 43.3%, but employment, perceived insufficient breast milk, and the number of children remain significant barriers to exclusive breastfeeding. Complementary feeding practices are not well documented, with only two studies found. The prevalence of stunting, wasting, and being underweight remains high among children under 5 years in the Gaza Strip, with risk factors including poor awareness of healthy diets, poverty, poor socioeconomic situations, urbanization, and lifestyle among communities, in addition to the political situation and the blockade. To address these issues, it is recommended to increase public awareness campaigns to promote healthy eating habits, provide training for healthcare providers on appropriate infant and young child feeding practices, improve maternal health, and address the underlying socioeconomic and political issues. It is also recommended to conduct more research on complementary feeding practices and monitor progress toward achieving the World Health Organization's nutrition goals.

The prevalence of overweight and obesity is high among children and adults in Palestine. Age, gender, living area, low physical activity, consumption of carbonated soft drinks and energy drinks, less consumption of milk products, and mothers' low education levels are significant predictors of overweight and obesity among children. Moreover, underweight children were found to be associated with households not having enough food for at least the last 2 days. Studies found a strong relationship between overweight and obesity and hypertensive disorders, diabetes mellitus, and lipid profiles. Overweight and obesity are also prevalent among adults, particularly women. The national data highlights the need for comprehensive interventions to control overweight and obesity, particularly among vulnerable populations. Such interventions should focus on encouraging healthy eating habits and physical activity and improving mothers' education levels. Furthermore, these programs should address the root causes of undernutrition to achieve sustainable development goals. Future research should focus on developing culturally appropriate interventions to address this public health problem in Palestine.

## Author contributions

Conceptualization: HAS, EA, and AA-J. Methodology, writing—original draft preparation, and visualization: EA and HAS. Review and editing: HAS. All authors have read and agreed to the published version of the manuscript.
